# Analysis of the Use of Extracorporeal Circulation on the In-Hospital Outcomes of Dialytic Patients Who Underwent Myocardial Revascularization Surgery

**DOI:** 10.5935/abc.20160180

**Published:** 2016-12

**Authors:** Matheus Miranda, João Nelson Rodrigues Branco, Guilherme Flora Vargas, Nelson Americo Hossne Jr, Michele Costa Yoshimoto, José Honorio de Almeida Palma da Fonseca, José Osmar Medina de Abreu Pestana, Enio Buffolo

**Affiliations:** 1 Hospital do Rim e Hipertensão - Universidade Federal de São Paulo - Escola Paulista de Medicina (UNIFESP-EPM) - Brazil; 2 Universidade Federal de São Paulo (UNIFESP), São Paulo, SP - Brazil

**Keywords:** Myocardial Revascularization, Extracorporeal Circulation / utilization, Clinical Evolution, Dialysis, Hospitalization

## Abstract

**Background:**

Myocardial revascularization surgery is the best treatment for dyalitic patients with multivessel coronary disease. However, the procedure still has high morbidity and mortality. The use of extracorporeal circulation (ECC) can have a negative impact on the in-hospital outcomes of these patients.

**Objectives:**

To evaluate the differences between the techniques with ECC and without ECC during the in-hospital course of dialytic patients who underwent surgical myocardial revascularization.

**Methods:**

Unicentric study on 102 consecutive, unselected dialytic patients, who underwent myocardial revascularization surgery in a tertiary university hospital from 2007 to 2014.

**Results:**

Sixty-three patients underwent surgery with ECC and 39 without ECC. A high prevalence of cardiovascular risk factors was found in both groups, without statistically significant difference between them. The group "without ECC" had greater number of revascularizations (2.4 vs. 1.7; p <0.0001) and increased need for blood components (77.7% vs. 25.6%; p <0.0001) and inotropic support (82.5% vs 35.8%; p <0.0001). In the postoperative course, the group "without ECC" required less vasoactive drugs, (61.5% vs. 82.5%; p = 0.0340) and shorter time of mechanical ventilation (13.0 hours vs. 36,3 hours, p = 0.0217), had higher extubation rates in the operating room (58.9% vs. 23.8%, p = 0.0006), lower infection rates (7.6% vs. 28.5%; p = 0.0120), and shorter ICU stay (5.2 days vs. 8.1 days; p = 0.0054) as compared with the group with ECC surgery. No difference in mortality was found between the groups.

**Conclusion:**

Myocardial revascularization with ECC in patients on dialysis resulted in higher morbidity in the perioperative period in comparison with the procedure without ECC, with no difference in mortality though.

## Introduction

Chronic renal failure is an independent risk factor for coronary diseases and their complications. The severity of injuries is inversely proportional to glomerular filtration rate and, for this reason, ischemic cardiovascular diseases are the main cause of mortality in these patients.^1,2^ In addition to uremia, other factors including poor quality of distal coronary bed, hyperhomocysteinemia, increased calcium-phosphorus product, oxidative stress and exacerbated inflammatory and atherosclerotic status are associated with coronary disease severity.^3-6^ Myocardial revascularization surgery has demonstrated higher long-term survival and lower risk for myocardial infarction and cardiovascular death as compared with coronary angioplasty in chronic renal failure patients on hemodialysis. However, surgical intervention still results in high morbidity and mortality in these patients.^7-11^

Myocardial revascularization surgery without extracorporeal circulation (ECC) has benefits when compared with its use, including lower inflammatory response, less embolization of atherosclerotic material, lower requirement for blood transfusion and vasoactive drugs, shorter time of mechanical ventilation and shorter stay in the intensive care unit (ICU).^12-14^ National and international articles have suggested an association between the use of ECC and postoperative morbidity and mortality.^15,16^

## Objectives

To evaluate the effect of the use of ECC on the in-hospital outcomes of patients with chronic renal failure on hemodialysis following myocardial revascularization surgery, trying to identify the best surgical strategy for this group of patients.

Medical records of 102 consecutive, unselected patients with chronic renal failure on dialytic therapy, who underwent myocardial revascularization surgery in a public tertiary university hospital in the period from 2007 to 2014 were assessed. Patients undergoing other concomitant procedures (e.g. heart valve, carotid, aortic surgeries) and patients with previous cardiac surgery were excluded. We assessed demographic, clinical and intraoperative data, and postoperative complications during patients' hospital stay. Preoperative risk was calculated by the *European System for Cardiac Operative Risk Evaluation II* (EuroSCORE II).^17^ The study group was divided into two subgroups ("with ECC" and "without ECC"). The study was approved by the local ethics committee.

### Surgical technique

Indication for myocardial revascularization surgery was based on national and international guidelines.^18-20^ Surgical planning was based on injuries detected by cineangiography, feasibility of surgical revascularization of distal coronary bed, and choice of the best vascular graft for each coronary artery. The choice of using or not using ECC was at the surgeon's discretion. In the operating room, central venous access was obtained, and invasive medication for blood pressure control, anesthetic monitoring and general anesthesia were performed. A 12-14 cm was made on the skin in the presternal region followed by a median sternotomy. Left internal thoracic artery was dissected and skeletonized, taking care to avoid the opening of the pleura, and used for revascularization of anterior interventricular artery. The other vascular graft used was the great saphenous vein directed to the other coronary beds, dissected through incisions in the medial side of the thigh. All patients underwent dialysis on the day prior to the surgery.

*Technique with ECC*: Heparin administration at 4mg/kg was performed before aortic and atrial cannulation (double-staged cannula). The ECC was established after an activated clotting time (ACT) greater than 480 seconds was confirmed. During cardiac arrest induced by aortic clamping, myocardial protection with intermittent antegrade cold blood cardioplegia was performed every 15 minutes.

*Technique without ECC*: heparin and ACT control were administered at 2 mg/kg 10 minutes before coronary occlusion. Distal anastomoses were performed using *Octopus*® (*Medtronic*, Inc.) vacuum stabilizers and with proximal occlusion of the arteries. The anastomoses were carried out by priority of arteries with total occlusion.

### Statistical analysis

Quantitative variables were expressed as means and categorical variables as percentage. The Mann-Whitney test was used for comparison of quantitative variables and the Fisher's exact test for comparison of categorical variables. The level of significance was set at 5%. The statistical tests were performed using the BioEstat 5.0 software.

## Results

A total of 102 patients were included in the study, 63 underwent myocardial revascularization surgery with ECC and 39 without ECC. Demographical and laboratory data are described in Tables 1 and 2, respectively. A high prevalence of cardiovascular risk factors was observed, with no statistically significant difference though. Three patients were under immunosuppressive and dialytic therapy due to kidney transplant rejection. Intraoperative data showed that patients operated with ECC had more revascularization of coronary arteries, required inotropic support and transfusion of blood derivatives more often, and used intra-aortic balloon pump in two occasions (Table 3).

**Table 1:** Demographic characteristics

Characteristics	With ECC n = 63	Without ECC n = 39	p
Age (years)	56.7	56.0	0.9316
Female sex (%)	22.2	30.7	0.3581
Time of dialysis (years)	2.9	4.4	0.8966
Hypertension (%)	100	100	1.0000
Diabetes (%)	80.9	66.6	0.1544
Dyslipidemia (%)	36.5	33.3	0.8325
Obesity (%)	12.6	15.3	0.5250
Smoking (%)	9.5	5.1	0.4837
Chronic obstructive pulmonary disease (%)	0	0	1.0000
Previous stroke (%)	14.2	12.8	1.0000
Previous angioplasty (%)	22.2	23.0	1.0000
Heart failure (%)	19.0	10.2	0.2757
Previous acute myocardial infarction (%)	15.8	20.5	0.5988
Peripheral arterial disease (%)	14.2	15.3	1.0000
Stable angina (%)	14.2	23.0	0.2923
Unstable angina (%)	11.1	12.8	0.9999
Left coronary trunk lesion (%)	9.5	15.3	0.5284
Preserved ventricular function (LVEF>50%)	85.7	71.7	0.1228
EuroSCORE II (%)	2.93	2.77	0.9784

ECC: extracorporeal circulation; LVEF: left ventricular ejection fraction.

**Tabela 2:** Dados laboratoriais pré-operatórios

Laboratory variable (mean)	With ECC n = 63	Without ECC n = 39	p
Creatinine (mg/dL)	7.8	8.2	0.5261
Urea (mg/dL)	110.9	120.3	0.1348
Hemoglobin (g/dL)	11.2	12.1	0.3350
Sodium (mEq/L)	135.8	135.4	0.6935
Potassium (mEq/L)	4.8	5.1	0.3578

ECC: extracorporeal circulation.

**Table 3:** Intraoperative data

Variable	With ECC n = 63	Without ECC n = 39	p
Number of anastomoses	2.4	1.7	<0.0001
Inotropic support (%)	82.5	35.8	<0.0001
Transfusion (%)	77.7	25.6	<0.0001
Intra-aortic balloon (%)	3.1	0	0.5230
Intraoperative death (%)	0	0	1.0000

ECC: extracorporeal circulation.

Data of postoperative course are found in Table 4. The most frequent complications were atrial fibrillation, infection and prolonged mechanical ventilation. Patients with ECC surgery had lower infection rate, required less vasoactive drugs and shorter time of mechanical ventilation and ICU stay, as well as greater success in weaning from mechanical ventilation in the operative room.

**Table 4:** Postoperative data

Complication	With ECC n = 63	Without ECC n = 39	p
Surgical reexploration (%)	3.1	2.5	1.0000
Postoperative infarction (%)	3.1	5.1	0.6356
Atrial fibrillation (%)	25.3	17.9	0.4686
Use of vasoactive drug (%)	82.5	61.5	0.0340
Time of use of vasoactive drug (days)	3.5	2.9	0.1915
Extubation in the operative room (%)	23.8	58.9	0.0006
Ventilation for more than 24 hours (%)	15.8	10.2	0.5583
Duration of mechanical ventilation (hours)	36.3	13.0	0.0217
Stroke (%)	1.5	5.1	0.5564
Infection (%)	28.5	7.6	0.0120
Rehospitalization within 30 days (%)	1.5	0	1.0000
Incision complications (%)	3.1	2.5	1.0000
Vasoplegia (%)	11.1	2.5	0.1498
In-hospital mortality (%)	12.6	5.1	0.3103
Time ICU stay (days)	8.1	5.2	0.0054
Time of hospital stay (days)	13.0	10.8	0.5921

ECC: extracorporeal circulation; ICU: intensive care unit.

## Discussion

Chronic renal failure is an independent factor for coronary heart diseases and culminates in higher risk of perioperative morbidity and mortality. The study group showed high prevalence of cardiovascular risk factors, such as hypertension, diabetes, dyslipidemia, and history of cardiovascular diseases, similar to those reported in the CHOICE study.^3^ With respect to these aspects, no statistically significant differences were detected between the groups, indicating that they were comparable in terms of demographic variables. However, the greater number of revascularizations performed in the group operated with ECC (p<0.0001) suggests higher severity of coronary disease (the SYNTAX Score could not be obtained from the available data), and hence a possible selection bias. This was predictable and understandable, since the technique without ECC hinders the revascularization of coronaries in the posterior territory, which explains the greater number of patients with three-vessel disease in the group with ECC surgery. Despite the greater number of diseased arteries in the ECC group, no difference between the groups in mortality risk was detected by the EUROScore II.

Patients operated without ECC required less inotropic support and blood transfusion during surgery (p<0.0001), similar to that reported by other authors [20]. In the postoperative course, patients without ECC surgery were extubated earlier, required less vasoactive drugs (82.5% vs. 61.5% p = 0.0340) and had lower infection rate (28.5% vs. 7.6% p = 0.0120) as compared with patients with ECC surgery. These data reflect the intense inflammatory response triggered by the passage of blood through non-endothelial surfaces of ECC, leading to vasoplegia, increased need for vasopressors and higher morbidity and mortality. In contrast to previously reported by other authors and by us, there was no statistically significant difference in in-hospital mortality between the groups, with a tendency of higher mortality in the group of patients that underwent surgery with ECC (12.6% vs. 5.1% p = 0.3103).^14-16,21^

The low mortality in the group without ECC is comparable to studies by Milani et al. and Fukushima et al., in which revascularization without ECC had low morbidity, without occurrence of death during hospital stay.^22,23^

## Conclusion

Myocardial revascularization surgery is feasible in patients on dialysis, despite higher morbidity and mortality rates as compared with the general population. As compared with the surgery without ECC, the use of ECC resulted in longer duration of mechanical ventilation and longer ICU stay, increased need for blood derivatives, and inotropic and vasoactive drugs, higher incidence of infection and lower extubation rate in the operating room. However, no statistically significant difference in mortality was detected between the groups. These results suggest that myocardial revascularization surgery without ECC in dialytic patients promotes lower mortality during hospitalization, without affecting short-term mortality.
